# Aberrant Fat Metabolism in *Caenorhabditis elegans* Mutants with Defects in the Defecation Motor Program

**DOI:** 10.1371/journal.pone.0124515

**Published:** 2015-04-07

**Authors:** Ming Sheng, Ava Hosseinzadeh, Somsundar Veppil Muralidharan, Rahul Gaur, Eva Selstam, Simon Tuck

**Affiliations:** 1 Umeå Center for Molecular Medicine, Umeå University, SE-901 87 Umeå, Sweden; 2 Department of Plant Physiology, Umeå University, SE-901 87 Umeå, Sweden; Brown University/Harvard, UNITED STATES

## Abstract

The molecular mechanisms by which dietary fatty acids are absorbed by the intestine, and the way in which the process is regulated are poorly understood. In a genetic screen for mutations affecting fat accumulation in the intestine of *Caenorhabditis elegans*, nematode worms, we have isolated mutations in the *aex-5* gene, which encodes a Kex2/subtilisin-family, Ca^2+^-sensitive proprotein convertase known to be required for maturation of certain neuropeptides, and for a discrete step in an ultradian rhythmic phenomenon called the defecation motor program. We demonstrate that *aex-5* mutants have markedly lower steady-state levels of fat in the intestine, and that this defect is associated with a significant reduction in the rate at which labeled fatty acid derivatives are taken up from the intestinal lumen. Other mutations affecting the defecation motor program also affect steady-state levels of triglycerides, suggesting that the program is required *per se* for the proper accumulation of neutral lipids. Our results suggest that an important function of the defecation motor program in *C*. *elegans* is to promote the uptake of an important class of dietary nutrients. They also imply that modulation of the program might be one way in which worms adjust nutrient uptake in response to altered metabolic status.

## Introduction

Fatty acids are integral constituents of all cells and have four distinct essential roles, to store energy, to act as building blocks for membrane phospholipids, to function as hormones regulating physiology or metabolism, and to act as donor molecules used in the modification of certain proteins. Although animals are able to synthesize fatty acids *de novo*, when dietary nutrients are available, most are thought to obtain a significant proportion from their diet. In addition, some animals (including all mammals) are unable to synthesize certain essential fatty acids, which must, therefore, be taken up. Despite the importance of dietary fatty acids for animals, however, important aspects of fatty acid uptake remain to be elucidated. In particular, the molecular mechanisms regulating the transport of fatty acids from the intestinal lumen across the lipid bilayer that constitutes the plasma membrane are not fully understood [1].

The majority of lipids in the diet are in the form of phospholipids or triglycerides. Unaided, phospholipids cross membranes bilayers only very slowly [2]. Dedicated phospholipid translocases have been identified that can catalyse the translocation of specific phospholipids from the exoplasmic to the cytosolic leaflet of membranes [2]. For example, *C*. *elegans* mutants lacking the TAT-1 phospholipid translocase are defective in their ability to take up phosphatidylserine from the intestinal lumen [3]. Triglycerides cannot cross membrane bilayers but must first be hydrolysed. While the mechanisms by which dietary triglycerides are emulsified and hydrolysed within the intestinal lumen of animals has been well characterized, exactly how the free fatty acids released following hydrolysis cross the plasma membrane of cells lining the lumen, and how this process is regulated are still unclear [1]. An understanding of intestinal fatty acid transport is important, in part because excessively fatty diets are associated with obesity and an increased incidence of numerous diseases including heart disease and type II diabetes mellitus.

*Caenorhabditis elegans* is a valuable model for investigating many aspects of fat metabolism including lipid transport [4, 5]. The core metabolic pathways by which fatty acids are synthesized or oxidized are conserved, and the rates of fatty acid synthesis and oxidation are modulated by proteins orthologous to those with the equivalent functions in mammals. *C*. *elegans* is able to synthesize all the fatty acids needed for its growth and development [5, 6]. However, at least when worms are fed with an excess of bacteria in laboratory environments, an appreciable proportion of lipids incorporated into worm tissue is derived from the diet and not synthesized [7]. For example, under such conditions, only 7% of palmitate (C16:0) results from *de novo* synthesis. Even those fatty acids required for normal physiology that are not present in *E*. *coli* (which is not thought to be the primary food source in *C*. *elegans* natural environments) can be taken up when supplied in the diet [8].

As they are in mammals, fatty acids are stored as triglycerides in specialized storage organelles that have been called fat granules or lipid droplets [4]. The main tissue in which triglycerides are stored is the intestine, which is a tube consisting of 20 large cells arranged in a single layer [9]. The intestine runs from the pharynx, the feeding organ, to the rectum and, like the intestines of larger animals including mammals, has microvilli facing the luminal (apical) surface though which nutrients are absorbed. Although the uptake of fatty acids is important for growth and reproduction in *C*. *elegans*, little is known either about the mechanism by which uptake occurs or the way in which it is regulated.

Rhythmic phenomena are observed in very many evolutionarily distinct types of organisms, and can occur with periods ranging from seconds to years. Ultradian rhythms, those that have periods of less than 24 h, are also common and are exemplified by such fundamental processes as the beating of the heart, peristalsis and breathing [10]. In *C*. *elegans*, an ultradian rhythm called the defecation motor program (DMP), which occurs every 45 s, has been studied intensely at a mechanistic level [11–13]. The program consists of three distinct muscular contractions, which occur in rapid succession, that ultimately result in part of the contents of the intestine being excreted [11, 12]. First, a group of muscles lining the body wall in the posterior part of the worm contract (the posterior body contraction, pBoc) and then relax causing the contents of the intestinal lumen to be pushed first up and then back down the lumen. About 1 s after the posterior muscles relax, anterior body wall muscles contract (the anterior body contraction, aBoc) driving the head of the animal backwards into the anterior part of the intestine. Finally, as the anterior body contraction reaches its maximum, enteric muscles contract resulting in expulsion (Exp) of some of the luminal material through the rectum. The pacemaker for these rhythmic contractions resides in the intestine itself and consists of the rhythmic release of Ca^2+^ from endoplasmic reticulum into the cytoplasm triggered by an inositol-1,4,5-trisphosphate receptor, ITR-1 [14, 15]. The pacemaker drives the three separate muscular contractions independently so that each contraction can occur in the absence of the others [12]. The pBoc is activated by PBO-4/NHX-7, a Ca^2+^-sensitive Na^+^/H^+^ exchange pump present in the plasma membrane of the posterior intestinal cells [16, 17]. Protons released into the pseudocoelomic space between the intestine and the posterior body wall muscles activate a proton-gated channel in the membranes of the muscle cells consisting of PBO-5 and PBO-6 [16]. Activation of the channel leads to depolarization of the muscle membrane and contraction. The aBoc and Exp parts of the cycle are controlled by a calcium-sensitive subtilisin-like proprotein convertase, AEX-5, which acts in the intestine along with AEX-4, to promote the maturation and secretion of neuropeptides including NLP-40 [12, 18, 19]. NLP-40 activates Exp by activating a G protein-coupled receptor, AEX-2, on AVL and DVB neurons causing them to release GABA, which in turn excites enteric muscles that control the opening of the rectum [12, 18, 19]. Although, the molecular mechanisms regulating the DMP are becoming well understood, the reason for the existence of the program has not been extensively addressed.

In order to understand better how fat accumulation is regulated in *C*. *elegans*, we have performed a genetic screen for mutations causing reduced triglyceride accumulation. Here we report on two mutations isolated in this screen, *sv75* and *sv76*, which we demonstrate are alleles of *aex-5*. We show by staining with lipid-binding dyes, and by analysis of lipids by gas-liquid chromatography that *aex-5* mutants have reduced steady-state levels of triglycerides. Other mutants affecting both the aBoc and Exp parts of the DMP also have reduced steady-state levels of triglyceride. Furthermore, the strength of the accumulation defect correlates with the severity of the DMP defect suggesting that the muscular contractions that occur during these parts of the program are required for proper triglyceride accumulation. Mutations affecting specifically the pBoc or Exp parts of the DMP also show defects in triglyceride accumulation but to a lesser extent. *aex*, *pbo* and *exp* mutants are all defective in rate at which they take up fluorescently labelled fatty acids from the intestinal lumen suggesting that the reduced steady state levels of fat are at least in part the result of a reduced ability to take up dietary fats.

## Materials and Methods

### Worm cultures

*Caenorhabditis elegans* strains were cultured on *E*. *coli* strain OP50 bacteria and manipulated as described [20]. Unless otherwise noted, all strains were cultured at 20°C. The following mutations and strains were used: *LG I*: JT9, *aex-1*(*sa9*); JT23, *aex-5*(*sa23*)*;* JT24, *aex-6*(*sa24*) [12]; VB0125, *unc-54*(*r293*); VB0524, *unc-101*(*m1*) (This study); CB406, *unc-57*(*e406*) [20]; CB0261, *unc-59*(*e261*) [21]; VB2379, *aex-5*(*sv76*); VB2485, *aex-5*(*sv75*) (This study); *LG II*: JT6, *exp-1*(*sa6*) [12]; *LG III*: JT7, *pbo-1*(*sa7*) [12]; *LG V*: EG4, *pbo-5*(*ox4*) [16]; JT47, *egl-8*(*sa47*) [12]; *LG X*: JT3, *aex-2*(*sa3*); JT5, *aex-3*(*sa5*); JT5244, *aex-4*(*sa22*) [12]; RB793, *pbo-4*(*ok583*); RB1379, *pbo-6*(*ok1564*) (Obtained from the *C*. *elegans* gene knockout consortium via the *Caenorhabditis* Genetics Center). CB4856 Hawaiian wild-type *C*. *elegans*.

### Isolation and mapping of *sv75* and *sv76*

*aex-5*(*sv75*) and *aex-5*(*sv76*) were isolated in forward genetic screens for mutants following ethylmethanesulfonate (EMS) mutagenesis. The mutants were backcrossed six times against the wild-type N2 strain before analysis. Single nucleotide polymorphism (SNP) mapping was used to map the mutations to the right arm of chromosome I. Two-point genetic mapping was performed with *unc-57*(*e406*), *unc-101*(*m1*), *unc-59*(*e261*) and *unc-54*(*r293*).

### Staining with lipophilic dyes

Published protocols were followed to stain fixed worms with Sudan Black [22], Oil Red O [23] or Nile Red [24]. Prior to staining with Nile Red or Oil Red O, worms were fixed with isopropanol as previously described [23, 24]. For Sudan Black staining, worms were fixed in paraformaldehyde and dehydrated in ethanol as described by McKay *et al*. [22]. To assess the uptake of fatty acids into the intestine, hermaphrodite worms were incubated for 10 minutes in M9 buffer containing a final concentration of 20 nmol/l C_1_-BODIPY 500/510 C_12_ (Invitrogen) (and 0.1% DMSO) [25]. Prior to microscopy, excess dye was removed by washing in M9 buffer.

### Lipid extraction and gas-liquid chromatography

The ratios of total triglycerides to total phospholipids in different strains were determined by first measuring the amounts of triglycerides and phospholipids relative to known amounts of added triglyceride and phospholipid standards containing C13:0 and C17:0 acyl chains respectively. The ratios were calculated as described previously [24]. Total lipids (containing both triglycerides and phopsholipids) were extracted by either of two published methods [24, 26]. Triglycerides and phospholipids were separated by column chromatography on HyperSep S 1 columns [24]. Triacylglyceride and phospholipid fractions eluting from the columns were collected and the solvents evaporated under a gentle stream of N_2_ gas. The lipids were converted to fatty acid methyl esters (FAMEs) by heating for 2 h at 80°C in 1 ml of a 5% solution of H_2_SO_4_ in dry methanol. Following esterification, the reactions were stopped by the addition of 1 ml of water and the lipids were extracted into 2 ml of petroleum ether 40–60 (VWR). After evaporation of the ether, the FAMEs were taken up in heptane and analyzed by gas chromatography on a Varian 3400 gas chromatograph.

### RNAi

RNAi of *aex-5* was performed by feeding worms bacteria harboring a plasmid encoding double-stranded RNA from part of the *aex-5* gene. The bacterial clone came from a library of clones received from the Ahringer laboratory [27].

### Confocal microscopy of Nile Red and C_1_-BODIPY 500/510 C_12_-stained worms

Fixed worms stained with Nile Red, and live whole worms stained with C_1_-BODIPY 500/510 C_12_ were viewed by fluorescence confocal microscopy performed with a Nikon A1 confocal microscope. To quantify C_1_-BODIPY 500/510 C_12_ uptake, ImageJ ROI Manager (National Institute of Health, Bethesda, MD) was used to measure the fluorescence intensity within the cytoplasm in a section at the center of the worm. The fluorescence intensity in two representative areas was measured for each worm. Six worms from each strain were analyzed.

### Lipid droplet determination

To determine the relative proportion of the worm occupied by lipid droplets, total volumes of droplets within a given region were determined by the analysis of 3-dimensional (3-D) images generated from multiple confocal fluorescent micrographs with the Imaris computer program (Bitplane, South Windsor, CT, USA). This software has been specifically developed for the visualization and analysis of 3-D and 4-D datasets. Confocal fluorescent images of multiple focal planes of Nile Red-stained stained worms were obtained at a resolution of 512 by 512 pixels using a 63x objective and 2.5x software magnification. The step size in the z dimension was 0.5 μm. The combined volume of the Nile Red-stained regions within approximately 1/3 of the worm was determined and compared to the total volume of the same region. For each strain, six different worms were analyzed and two different regions were examined for each worm. Imaris software version x64 7.6.5 was used for the analysis.

### *fat-3* rescue experiments

Plates containing γ -linolenic acid (C18:3n6) were prepared as previously described [8]. To assay for rescue of *fat-3*, newly hatched L1 hermaphrodite larvae were placed onto plates supplemented with the fatty acid, incubated for different periods of time, and then transferred to plates lacking the supplement. Worms were assayed during the L4 and adult stages for rescue of the locomotion defect.

### Defecation cycle assay

To assess the DMP in *sv75* and *sv76* mutants, individual animals on standard culture plates were followed under the dissecting microscope through 9 cycles. The occurrence of pBocs, aBocs and Exps, as defined previously [12], were noted.

### Statistical analysis

Statistical analyses were carried out with the computer software packages StatPlus:mac version 5.8.5.6 and GraphPad Prism for Mac version 6.

## Results

### *sv75* and *sv76*, two mutations causing reduced fat accumulation

At low magnification, *C*. *elegans* mutants containing increased levels of triglycerides have a darkened appearance caused by the increased refractivity of the fat storage granules in the intestine [28]. Conversely, in animals with reduced levels of triglycerides, the intestine is much lighter in appearance [22]. To identify mutations causing changes in triglyceride accumulation in *C*. *elegans*, we carried out genetic screens for mutants with lightened intestines. Two of the mutations we isolated, *sv75* and *sv76*, appeared to affect the same gene: they mapped to close to one another, and failed to complement one another. All animals homozygous for either mutation or *trans*-heterozygous for the two alleles were markedly lighter in color than wild type.

**Fig 1:**
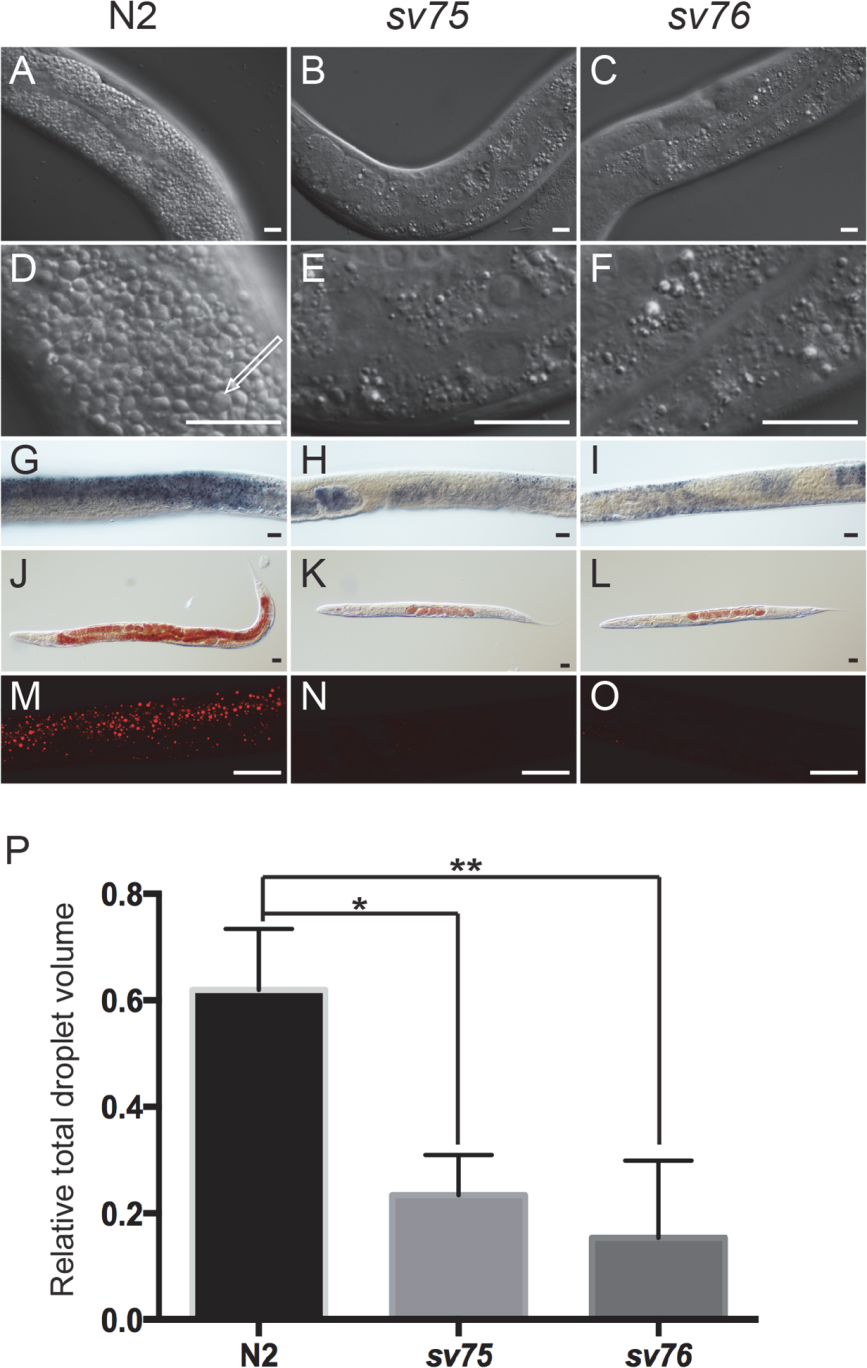
*sv75* and *sv76* cause decreased fat accumulation in the intestine. Representative micrographs of parts of young adult hermaphrodite worms viewed with differential interference contrast (DIC) (A-L) or fluorescence (M-O) optics. Scale bars are 10 μm. A-F show parts of the intestines of living worms; G-I show fixed worms stained with the lipophilic dye, Sudan Black. J-L show fixed worms stained with the lipophilic dye, Oil Red O. M-O show fixed worms stained with the fluorescent lipophilic dye, Nile Red. The arrow in D indicates a lipid droplet. P shows quantification (in arbitrary units) of the combined volume of Nile Red-stained droplets relative to tissue volume in wild type and in the mutants (Materials and Methods). Error bars indicate standard errors of the means. * and ** indicate significant differences between the means determined by one-way ANOVA (σ = 0.05) and Dunnett's multiple comparisons test.

**Fig 2:**
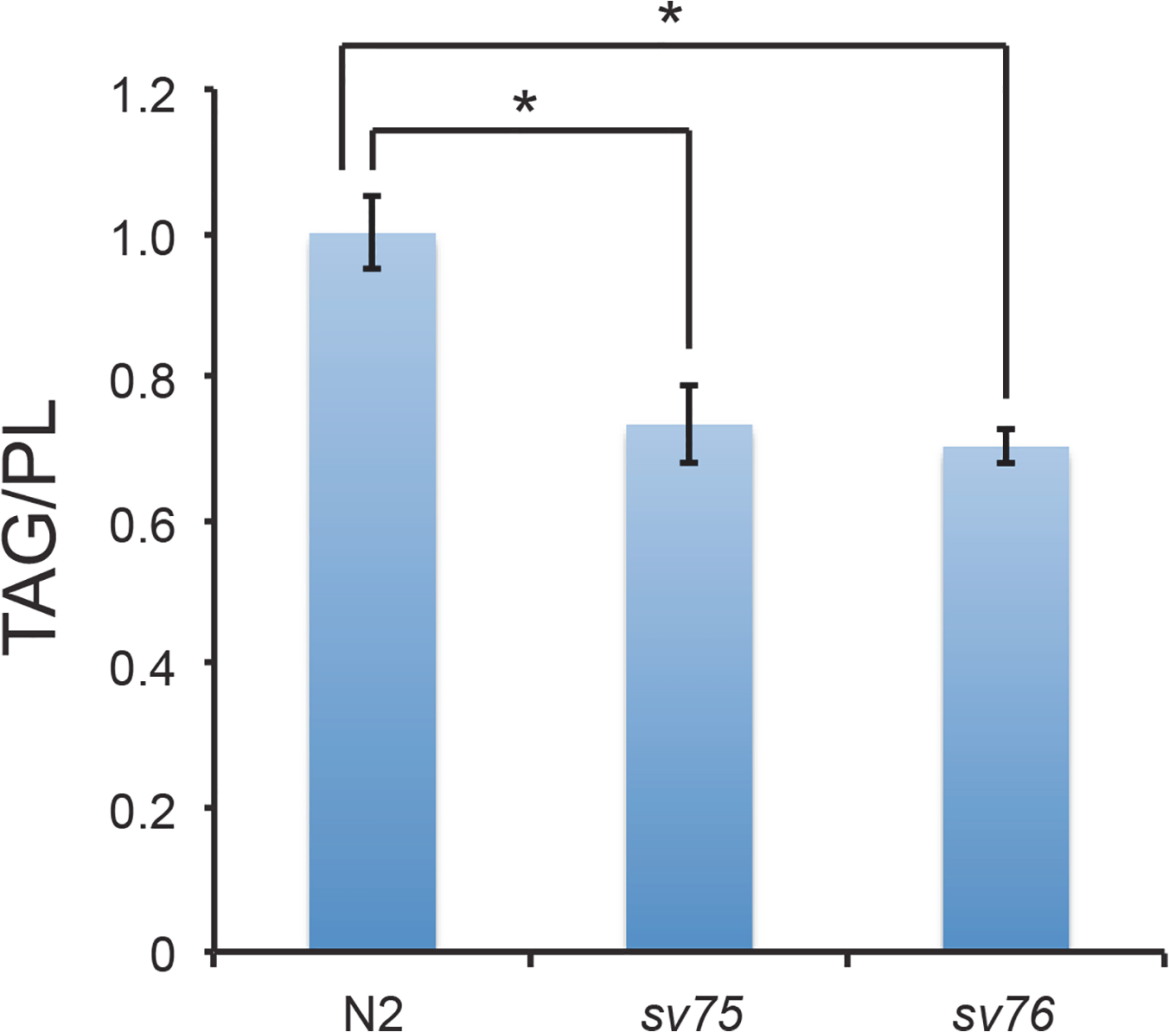
*sv75* and *sv76* mutant worms have lower triglyceride/phospholipid ratios than the N2 wild-type strain. The graph shows normalised ratios of total triglycerides (TG) to total phospholipids (PL) in extracts from young adult hermaphrodites. Levels of triglycerides and phospholipids relative to C13:0 and C17:0 standards respectively were determined by gas-liquid chromatography. Three extracts were examined for each genotype. Error bars represent 95% confidence intervals. * Indicates significant difference in the means determined by one-way ANOVA and Bonferroni test for differences between means (σ = 0.05).

Several observations confirmed that the intestines in *sv75* and *sv76* mutant animals did indeed contain less fat. First, the intestine was radically different in appearance in the mutants and was much less granular than in wild type (Fig 1A–1F). In wild-type worms, at 1000-fold magnification, two different types of granule are visible in intestinal cells, lysosome-related organelles (LROs) and a second type, in which triglycerides are predominantly stored, termed fat granules or lipid droplets [23, 29]. In living animals, the two types of granule can be distinguished by light microscopy in two ways. LROs autofluoresce when illuminated with blue light whereas the lipid droplets do not [29]. LROs are unevenly refractile whereas lipid droplets appear smooth when viewed with differential interference contrast (DIC) optics [29]. *sv75* and *sv76* mutants did not have reduced numbers of autofluorescent LROs S1 Fig However, the lipid droplets appeared to be much smaller and less numerous (Fig 1A–1F). When the mutants were stained with the lipophilic dyes Oil Red O or Sudan Black, which stain lipid droplets in *C*. *elegans* tissues including the intestine [23, 28], staining was invariably much less intense than that of wild type (Fig 1G–1L). Staining of the mutants with the fluorescent lipophilic dye, Nile Red was also considerably less intense (Fig 1M–1O). Measurement of the proportion of worm tissue occupied by the Nile Red-stained droplets revealed that it was much lower in the mutants compared to wild type (Fig 1P) S2 Fig Quantification of lipids extracted from the mutants by gas-liquid chromatography revealed a marked reduction in the ratio of triglycerides to phospholipids (Fig 2). Together, these results demonstrate that *sv75* and *sv76* mutant worms have significantly less steady-state levels of neutral fat in the intestine.

### *sv75* and *sv76* are alleles of *aex-5*, which is required continuously for normal fat levels

**Fig 3:**
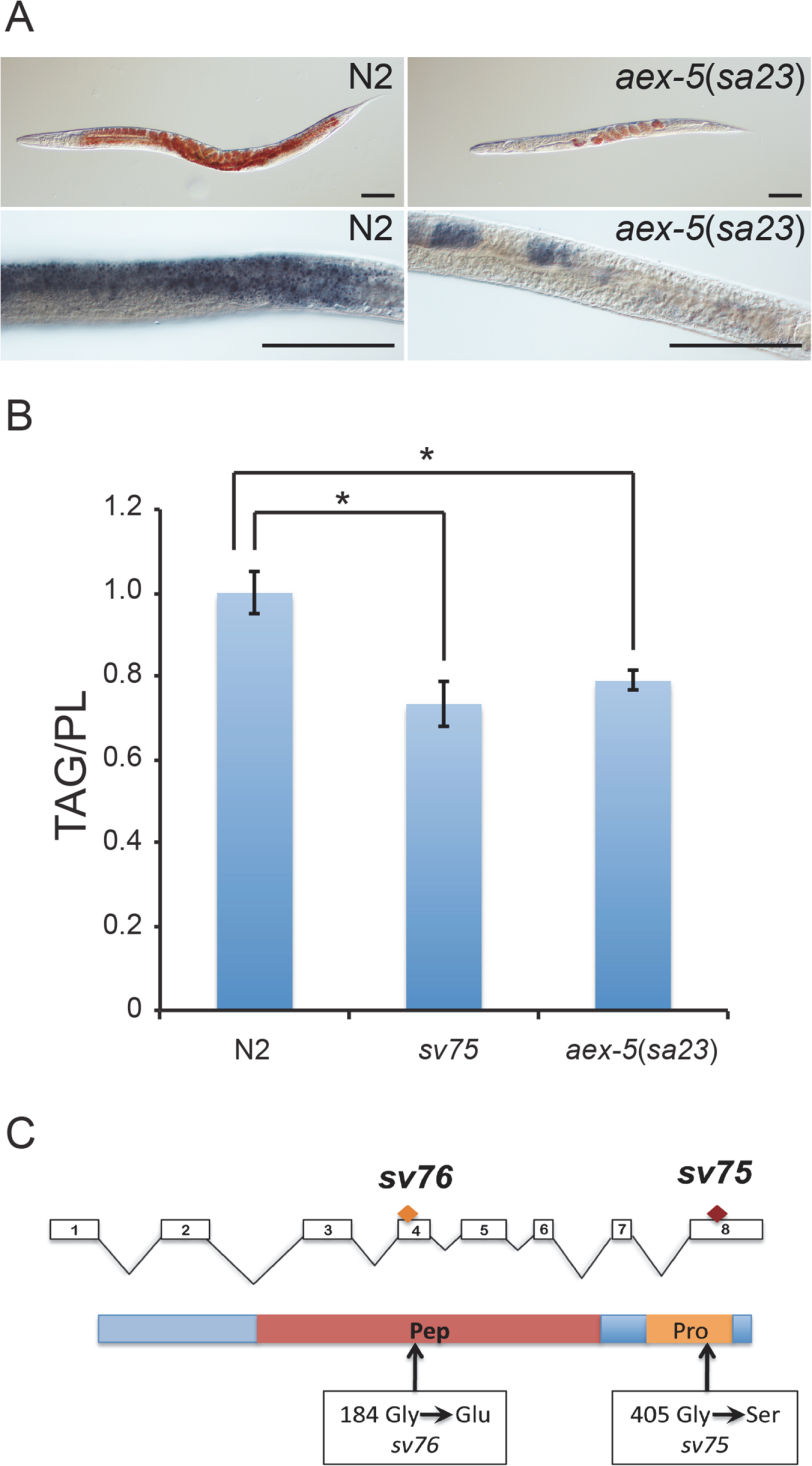
*sv75* and *sv76* are alleles of *aex-5*. A. Micrographs of fixed young adult hermaphrodites stained with Oil Red O (top panels) or Sudan Black (bottom panels). In wild-type (N2) worms, the dyes stain the intestine and the oocytes within the gonad. In the *aex-5*(*sa23*) mutant, only the oocytes show significant staining. Scale bars are 10 μm. B. Graph showing triglyceride/phospholipid ratios determined by gas-liquid chromatography. Error bars represent 95% confidence intervals. * Indicates significant difference in the means determined by one-way ANOVA and Fischer's test for least significant difference (σ = 0.05). C. Schematic diagram of the *aex-5* gene (at top) and protein (at bottom). White boxes and lines at top represent exons and introns respectively. The positions of the codons and amino acids affected by the *sv75* and *sv76* mutations are marked.

**Fig 4:**
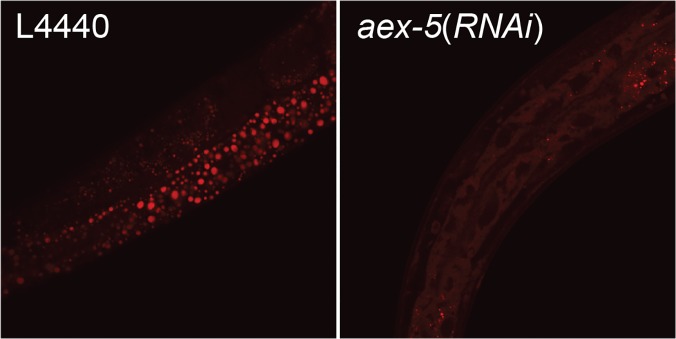
Fluorescence confocal micrographs of the intestines of young adult worms stained with Nile Red in isopropanol. The worms were subjected to RNAi by feeding after they had become adults. L4440 is the negative control in which wild-type adult worms were fed bacteria containing the plasmid vector L4440 alone. The *aex-5*(*RNAi*) worms were fed bacteria expressing double-stranded RNA from part of the *aex-5* gene.

Genetic mapping of *sv75* and *sv76* revealed that they mapped to within 0.1 map units of the *unc-54* gene (Materials and Methods). We knocked down the activities of genes lying close to *unc-54* and found that RNAi of *aex-5* caused a lightened intestine phenotype very similar to that seen in the mutants. An existing allele of *aex-5*, *sa23*, also displayed this defect. Furthermore, *aex-5*(*sv23*) hermaphrodites showed reduced staining with Oil Red O and Sudan Black (Fig 3A), and had a reduced relative amounts of triglycerides compared to phospholipids (Fig 3B). *sv75* and *sv76* both displayed highly penetrant defects in the DMP similar to those seen in the existing *aex-5* mutants [12]. In eight animals of each genotype observed over nine cycles, pBocs occurred normally but no aBocs or Exps occurred. In eight wild-type animals followed over the same period, 72 aBocs occurred and 71 Exps. DNA sequence analysis revealed that *sv75* and *sv76* are both associated with changes in the *aex-5* gene coding region that are predicted to introduce non-conservative changes into the amino acid sequence of the AEX-5 protein (Fig 3C). Adult animals subjected to RNAi of *aex-5* for 8 h had diminished levels of Nile Red staining (Fig 4) implying that *aex-5* is required continuously for proper triglyceride accumulation *i*.*e*. that the reduction in triglyceride in the mutants is not the result of a developmental defect. Thus, *sv75* and *sv76* are alleles of *aex-5*, which functions continuously to promote the accumulation of neutral fat.

### *aex-5* mutants ingest food normally and are able to synthesize fatty acids

*C*. *elegans* worms that are unable to ingest food properly because of defects in the feeding organ, the pharynx, are light in colour and have reduced levels of triglycerides [22]. However, earlier observations made on *aex* mutants, including those in *aex-5*, indicated that they do not have a reduced ability to ingest food [12]. Instead, they continue to ingest food normally until the intestine becomes filled with bacteria, after which time defecation occurs apparently by a passive mechanism, often independently of the DMP [12]. We quantified the rate of feeding in the *sv75* and *sv76* mutants and confirmed that they are not defective in the ingestion of food into the intestine. First, the rate of pharyngeal pumping (the rhythmic muscular contraction of the feeding organ that results in ingestion) was not reduced in the *sv75* and *sv76* mutants S3 Fig Second, the mutants were able to ingest bacterium-sized fluorescent beads normally S3 Fig

When early larvae are deprived of food, worms exit the reproductive life cycle and develop instead into dauer larvae, which accumulate triglycerides in lipid droplets in the intestine and other tissues. Much of the fatty acid within triglycerides stored in these animals is synthesized *de novo* rather than being taken up [30]. *aex-5* dauer larvae did not have obviously less fat than in wild type implying that *aex-5* is not required *per se* for the synthesis of fatty acids or their storage as triglycerides S3 Fig

### *aex-5* mutants display reduced uptake of a fluorescently-labelled fatty acid derivative

**Fig 5:**
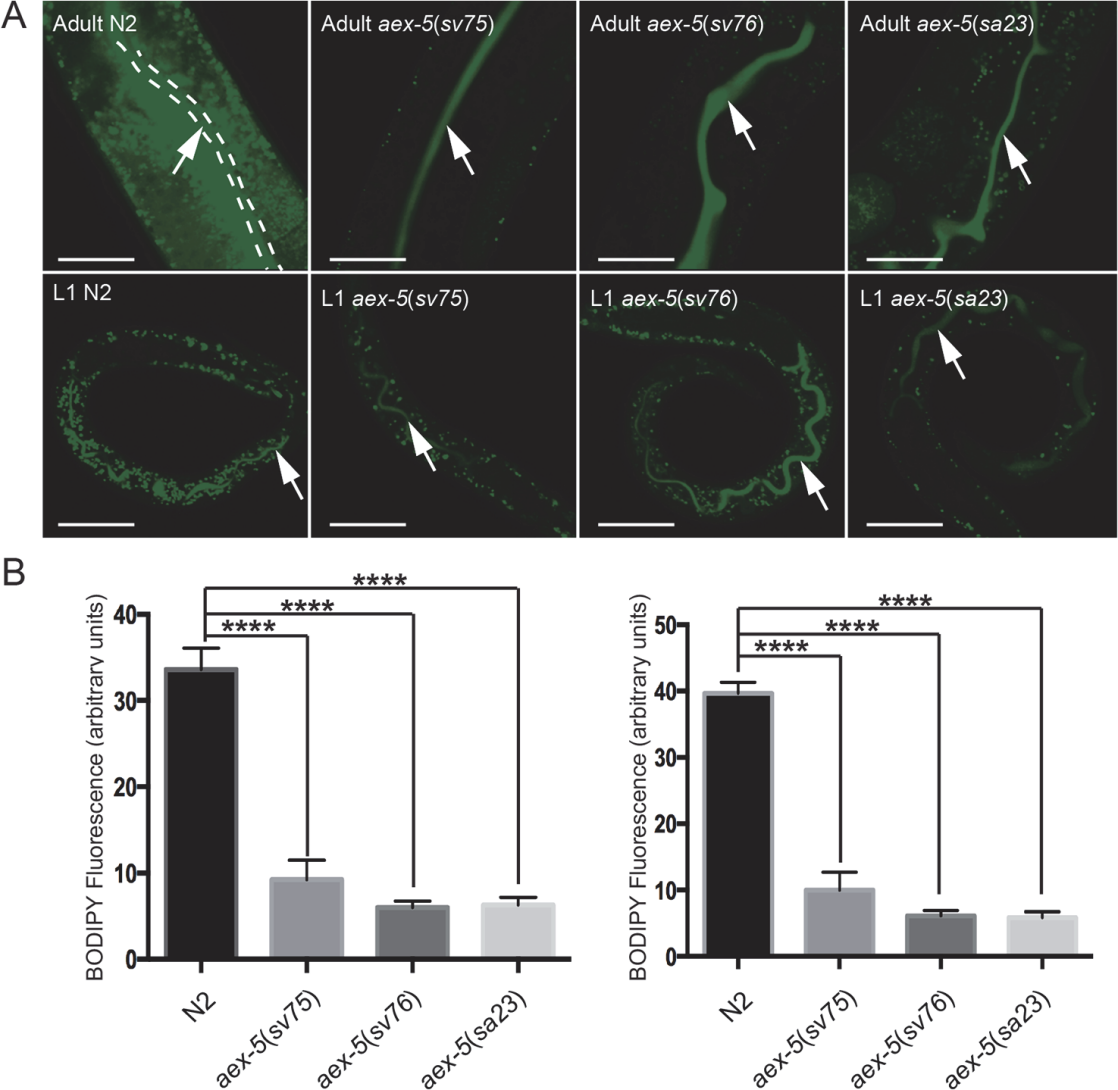
*aex-5* mutants take up a fluorescently labelled fatty acid derivative from the intestinal lumen less efficiently than wild type. A. Confocal fluorescence micrographs of worms fed the fluorescently labelled fatty acid derivative C_1_-BODIPY 500/510 C_12_. The panels at the top show parts of the intestines of adult worms; those at the bottom show first larval stage (L1) worms. The arrows point to the intestinal lumen where most of the dye is found in the mutants. Scale bars are 20 μ m. B. Graphs showing quantification (in arbitrary units) of the uptake of the dye into the cytoplasm of intestinal cells (Materials and Methods). Error bars indicate standard errors of the means. **** indicates significant difference in the means determined by one-way ANOVA and Dunnett's multiple comparisons test (σ = 0.05).

The reduction in the steady state levels of triglycerides in non-dauer worms with defects in the DMP could in principle be the result of increased utilization of triglycerides, a reduced ability to synthesize them, or by a reduction in the uptake of fatty acids or other metabolites from the lumen of the intestine. To examine whether the mutants had a reduced ability to take up fatty acids, we fed worms a fluorescently labelled free fatty acid derivative, C_1_-BODIPY 500/510 C_12_, and monitored the efficiency with which it was taken up from the lumen. In the C_1_-BODIPY 500/510 C_12_ molecule, the fluorescent group (4,4-difluoro-5-methyl-4-bora-3a,4a-diaza-*s*-indacene-3) is attached to the C1 carbon atom of a linear carbon chain with a carboxylic acid group on the C12 carbon atom. C_1_-BODIPY 500/510 C_12_ has been used extensively for studies on lipid trafficking in cells from many different organisms. When fed to live worms, C_1_-BODIPY 500/510 C_12_ accumulates within LROs within the cytoplasm of intestinal cells rather than in lipid droplets [23]. This observation suggests that LROs might act to protect intestinal cells from the detergent effects of free fatty acids by sequestering them until they are activated by esterification either for transport into mitochondria or for incorporation into triglycerides within the membrane of the endoplasmic reticulum. The observation is also consistent with previous work, which has shown that lipid droplets contain triglycerides rather than free fatty acids [31]. Whereas in wild-type worms, the dye is rapidly taken up into cytoplasm of intestinal cells, in the mutants, it mostly remained in the lumen (Fig 5A and 5B). Both adult worms and L1 larvae displayed the defect. The fluorescence intensity of the dye within the cytoplasm of intestinal cells in the mutants was less than 1/3 that in the intestinal cells in wild type (Fig 5B). Thus, the reduced steady-state levels of triglycerides in *aex-5* mutants are associated with a reduced rate of uptake of a fluorescently labelled free fatty acid.

One caveat with the experiments with C_1_-BODIPY 500/510 C_12_ is that the fluorescent group attached to the fatty acid backbone might affect transport, and that C_1_-BODIPY 500/510 C_12_ might not be transported in the same way as an unmodified fatty acid. Therefore, as a second test to assess the efficiency with *aex-5* mutants take up fatty acids we investigated rescue of *fat-3* locomotion defects by exogenously added naturally occurring poly unsaturated fatty acid (PUFA). *fat-3* encodes a Δ endesaturase required for the formation of C20 PUFAs [8]. *fat-3* mutants are viable but show a fully penetrant Unc phenotype that is efficiently rescued when worms are grown on plates supplemented with exogenously supplied PUFAs [8]. We found that whereas when newly hatched L1 larvae from a *fat-3*(*wa22*) single mutant strain were incubated on plates supplemented with γ-linolenic acid (C18:3n6) for 36 hours (and then returned to unsupplemented plates), they were all rescued for the Unc phenotype (n = 146). In contrast, when newly hatched *aex-5*(*sv75*); *fat-3*(*wa22*) or *aex-5*(*sv76*); *fat-3*(*wa22*) double mutant larvae were incubated on plates supplemented with C18:3n6 for the same length of time, all were Unc (and remained Unc for the rest of their development) (n = 132 and 160 respectively). *aex-5* single mutants are not uncoordinated (Thomas, 1990); indeed, *aex-5*(*sv75*); *fat-3*(*wa22*) and *aex-5*(*sv76*); *fat-3*(*wa22*) double mutants were efficiently rescued for the *fat-3* Unc phenotype when larvae were grown on plates containing C18:3n6 for longer (>60 hours) periods of time. Thus *aex-5* mutations reduce the efficiency with which exogenously supplied PUFA can rescue the *fat-3* mutant, a result that is consistent with the possibility that *aex-5* mutations reduce the efficiency with which free fatty acids are taken up.

### Other defecation mutants also have reduced lipid uptake and accumulation

**Fig 6:**
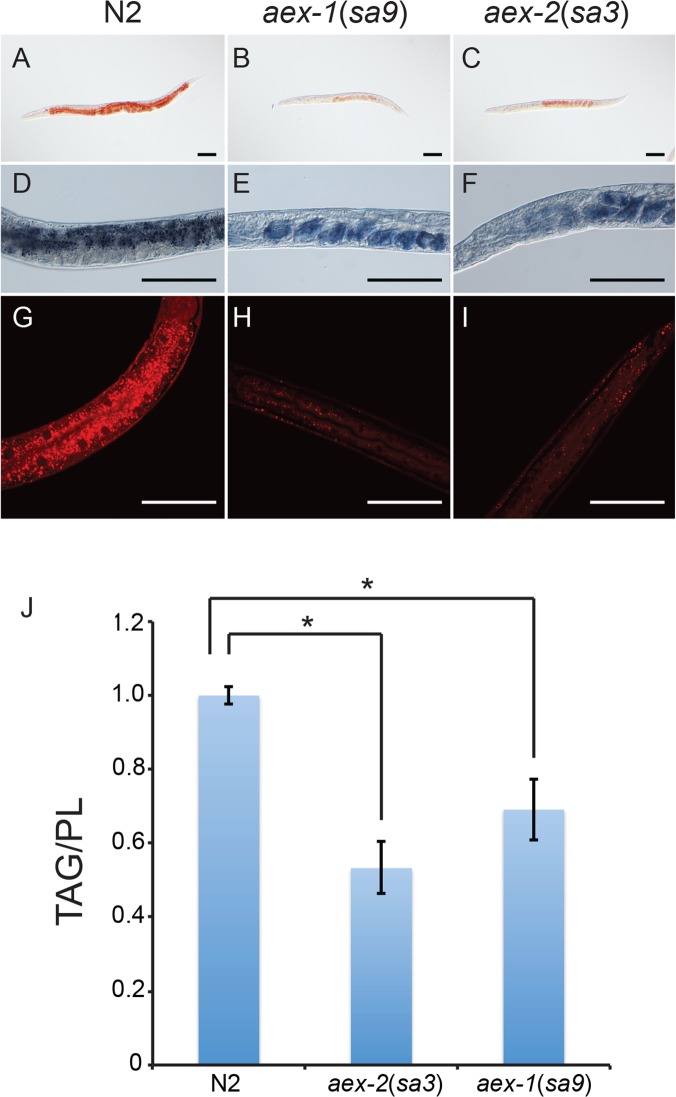
Mutations in *aex-1* and *aex-2* also cause a reduction in fat accumulation. A-C. Fixed, young adult hermaphrodite worms stained with Oil Red O. D-F. Micrographs showing part of the intestines of fixed, young adult hermaphrodite worms stained with Sudan Black. The staining in E and F is of eggs within the gonad. G-I. Fluorescence confocal micrographs of parts of young adult hermaphrodite worms fixed with isopropanol and stained with Nile Red. J. Graph showing the normalized ratios of total triglycerides to total phospholipids extracted from young adult hermaphrodite worms. Error bars represent 95% confidence intervals. * Indicates significant difference in the means determined by one-way ANOVA and Fischer's test for least significant difference (σ = 0.05).

**Fig 7:**
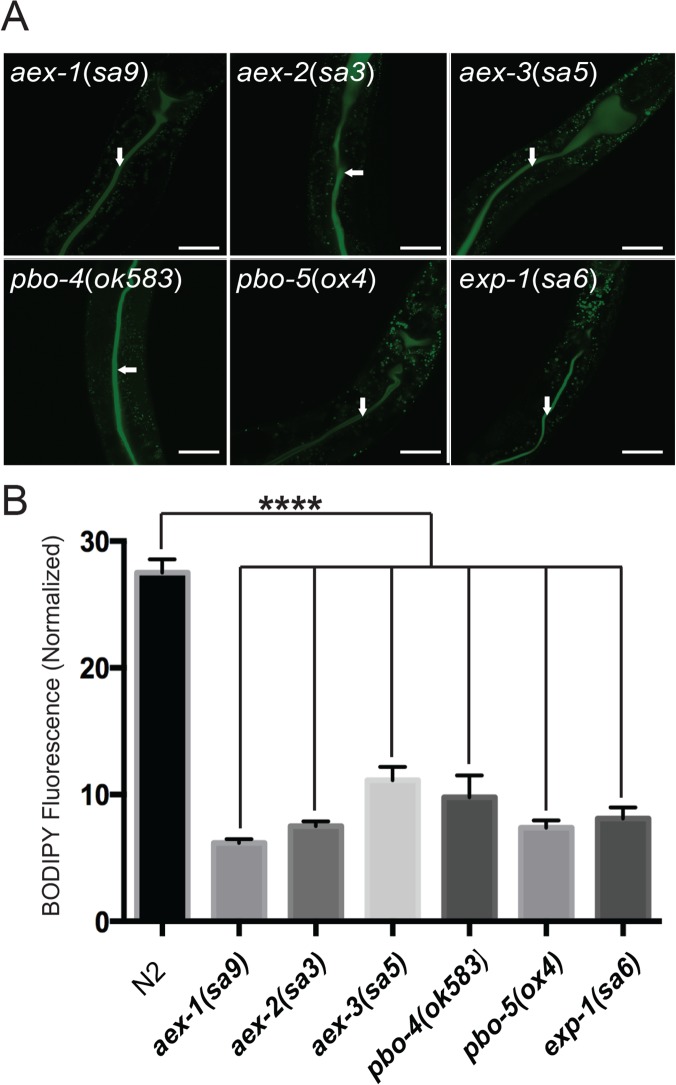
Other defecation mutants also display reduced uptake of C_1_-BODIPY 500/510 C_12_. A. Confocal fluorescence micrographs of worms fed the fluorescently labelled fatty acid derivative C_1_-BODIPY 500/510 C_12_. The arrows indicate the lumens of the intestine. In all panels, the anterior part of the intestine is up and (in most) slightly to the right. Scale bars are 40 μm. B. Quantification of the uptake of the dye into the cytoplasm of intestinal cells (in arbitrary units). Error bars indicate standard errors of the means. **** indicates significant difference in the means determined by one-way ANOVA and Dunnett's multiple comparisons test (σ = 0.05).

The genes required for the defecation motor program encode proteins that act in at least three different tissues and that have widely diverse biochemical functions. To help determine whether parts of the defecation motor program *per se* are required for normal triglyceride accumulation, or whether *aex-5* might act to promote triglyceride accumulation independently of its effects on the DMP, we examined fat accumulation in mutants lacking other components of the mechanism regulating the DMP. Like those in *aex-5*, mutations in *aex-1*, *aex-2*, *aex-3*, *aex-4* and *aex-6* affect the aBoc and Exp parts of the cycle causing it either to fail to occur at all or to occur less often [12]. Mutations in *aex-1*(*sa9*) and *aex-2*(*sa3*) mutants, in which the aBoc and Exp parts of the program are completely disrupted [12], displayed severely reduced staining with lipophilic dyes (Fig 6A–6I), markedly lower levels of triglycerides relative to phospholipids (Fig 6J), and reduced uptake of rate of C_1_-BODIPY 500/510 C_12_ uptake (Fig 7). *aex-3*(*sa5*), *aex-4*(*sa22*) and *aex-6*(*sa24*) mutants, which display slightly less severe DMP defects [12], also showed reduced staining with lipophilic dyes although the defects tended to be weaker than those of the *aex-1*, *aex-2* and *aex-5* mutants (Fig 7) S4 and S5 Figs Mutations in *exp-1*, which encodes a GABA-activated cation channel, specifically affect the Exp part of the program [12, 32]. An *exp-1* mutant showed reduced staining with Oil Red O, Sudan Black and Nile Red S4 and S5 Figs and reduced uptake of C_1_-BODIPY 500/510 C_12_ (Fig 7) suggesting that disruption of the Exp part of the program is sufficient to reduce steady-state levels of triglycerides and the rate of fatty acid uptake. However, the *exp-1* lipid defects were generally somewhat weaker than those observed with the *aex-1*, *aex-2* and *aex-5* mutants suggesting that the defects in the latter are caused by effects on both the aBoc and Exp parts of the program.

Mutations affecting the pBoc part of the cycle also displayed reduced staining by lipophilic dyes S4 and S5 Figs although the reduction was not as marked as that of the strongest *aex* mutants even in pBoc mutants homozygous for deletion mutations that are thought to eliminate gene activity. Measurement of lipid content by gas chromatography showed that *pbo-1* mutants had reduced amounts of triglycerides relative to phospholipids S4 Fig The mutants also displayed reduced uptake of C_1_-BODIPY 500/510 C_12_ (Fig 7). An *egl-8* mutant, which displays normally timed but weak pBocs [12], also showed lipid defects (Fig 7) S4 and S5 Figs It should be noted, however, that since *egl-8* mutations also affect feeding [33], the defects on lipid levels in this mutant might not solely be the result of defects in the defecation motor program.

## Discussion

We report here on the isolation and characterization of two mutations in the *aex-5* gene, *sv75* and *sv76*, identified in a genetic screen for mutations affecting fat metabolism. We demonstrate by a variety of methods that *aex-5*(*sv75*) and *aex-5*(*sv76*) homozygous mutant animals have reduced steady-state levels of triglycerides and that an existing *aex-5* mutant and *aex-5*(RNAi) animals also shows this defect. The mutants are not defective in feeding. Furthermore, *aex-5* dauer larvae accumulate fat normally suggesting no absolute requirement for *aex-5* for the synthesis or storage of fatty acids. The mutants do, however, have markedly reduced uptake of a fluorescently labelled free fatty acid from the lumen of the intestine. The lipid accumulation and uptake defects result from a continuous requirement for AEX-5 function rather than from a developmental defect.

The AEX-5 proprotein convertase has been shown to be required for the proper maturation of a number of different neuropeptides in *C*. *elegans* [34]. Since some neuropeptides are known to be able to affect metabolic pathways directly, in principle, AEX-5 might affect triglyceride accumulation by promoting the maturation of a neuropeptide that affects metabolism independently of the DMP. While our work does not exclude this possibility, it is noteworthy that all the DMP mutants we examined displayed defects in triglyceride accumulation. *aex-1* and *aex-2* mutants, which display similar DMP defects to those displayed by *aex-5* mutants, show defects in triglyceride accumulation that are at least as strong as those displayed by *aex-5* mutants. The finding that triglyceride levels are reduced in *aex-2* mutants, in which the secretion of NLP-40 occurs normally [19], suggests that disruption of the defecation motor program is sufficient to affect lipid levels. Furthermore, mutations that affect genes controlling pBoc or Exp also lead to reduced triglyceride accumulation. For these reasons, we favour a model in which *aex-5* mutations affect fat accumulation via their affects on the DMP.

Previous authors have noted that the mutations identified in the initial screens for mutants with defects in the DMP do not block feeding [12]: there appears to be no feedback mechanism to stop feeding when defecation does not occur. Indeed, since most of the DMP mutants were identified by virtue of their constipated phenotypes [12], their ability to feed was a necessary prerequisite for their isolation [worms that don’t feed cannot become constipated]. Our observations that both pharyngeal pumping and the ingestion of fluorescent beads into the intestinal lumen is not reduced in *aex-5* mutants lends further weight to the idea that defects in the DMP do not inhibit feeding. With the exception of relatively brief periods of quiescence, when feeding is much reduced, in the presence of bacteria of the strain *E*. *coli* OP50 (the standard food source used in *C*. *elegans* laboratories), worms feed constitutively [35, 36]. Under these conditions, a reasonable assumption is that the rate at which materials enters the intestinal lumen is approximately constant. Thus, as noted previously [37], since the DMP regulates the rate at which material leaves the intestine, the DMP effectively regulates the average volume of the intestinal lumen, and the average amount of time that material stays in the lumen. One possible explanation for our results, therefore, is that the efficiency with which free fatty acids enter intestinal cells is reduced when the luminal volume is enlarged. It has been suggested that the defecation volume is controlled by a combination of the three motor steps, pBoc, aBoc and Exp, and that the ability of control defecation volume precisely has adaptive value [37]. The ability to take up fatty acids and other nutrients efficiently would likely be advantageous.

While free fatty acids are able to pass through synthetic membrane bilayers by diffusion [1], it is not known whether cells in the intestine or in other tissues rely on this mechanism. In cases where the process has been investigated in depth, the transport of fatty acids into cells that are dependent upon fatty acids for their metabolism is heavily reliant upon protein carriers [38]. Cardiac myocytes, for example, are thought to take up at least 50–60% of the fatty acids they require via a protein carrier, FAT/CD36 [38]. Other proteins, including those in the fatty acids transport protein family (FATP1-6) and the plasma membrane isoform of fatty acid binding protein (FABPpm), can also promote the transport of fatty acids across membranes. Although no roles for their products have yet been established in fatty acid uptake in *C*. *elegans*, several genes encoding putative fatty acid translocases (including CD36) are present in the *C*. *elegans* genome [4]. It is possible that when the intestinal lumen is enlarged, either the passive diffusion of fatty acids or their transport protein-aided movement through the plasma membrane is compromised. Although we have not investigated the transport of other metabolites, we cannot exclude the possibility that the uptake of other nutrients is defective in defecation mutants. Thus, the mutants might have less fat in part because stored triglycerides are hydrolysed and oxidized to generate metabolites that can compensate for reduced amounts of these other nutrients.

While the volume of the lumen *per se* might affect the efficiency with which fatty acids or other metabolites are absorbed, it is also possible is that it is not so much the volume itself that is important but the thorough mixing of the luminal contents. In this case, the reduced uptake of C_1_-BODIPY 500/510 C_12_ we have observed in the DMP mutants might be because the contents of the lumen are not properly mixed or not properly distributed. As it is in other organisms, the lumen of the intestine in *C*. *elegans* is acidic [39]. Moreover, reduction in the activity of VHA-6, a proton pump, is associated with an increase in the luminal pH, and reduced steady-state levels of fat [39]. Besides pumping protons across the basolateral membrane of the posterior intestine, PBO-4/NHX-7 is also expressed on the apical surface of the posterior intestinal cells and is required for proper distribution of acidity within the lumen [40]. Imaging of living worms fed with acid-activated fluorophores has revealed that, in wild-type animals, an area of low pH forms periodically in the posterior part of the intestinal lumen [40]. This low-pH “hot spot” migrates over the course of a few seconds to the most anterior part of the intestine before moving towards the posterior again and dissipating. In *pbo-4* mutants, the hot spot fails to form properly and the anterior lumen does not become acidified [40]. Although it has not been demonstrated, the expectation is that in *pbo-5* mutants, the posterior lumen becomes acidified but, in the absence of the pBoc, the low pH hot spot fails to migrate. In *aex* mutants, one would expect the movement of the hot spot back down the lumen would be defective. Since the pKas of fatty acids are around 4.9, and the luminal pH has been estimated to be 4.4 [39], even subtle changes in average pH of the lumen, or in the distribution of protons, might be expected to change the chemical properties of free fatty acids in the lumen. A reasonable expectation is that the acid (unionized) forms of fatty acids, since they are much less polar, would pass through membranes more easily that the anionic forms, at least by passive diffusion. We have attempted to rescue the uptake defects of the DMP mutants by supplying the fatty acid derivative in buffers with reduced pH. We observed no rescue in these experiments but, since reducing the pH of the buffer surrounding the entire worm is a somewhat crude intervention, the absence of rescue does not rule out the possibility that the defects are, at least in part, caused by altered pH distribution. Previous workers have reported that the luminal pH does not change significantly when worms are fed buffers with altered pH [39], which may explain why no rescue was observed in our experiments.

Recent work has shown that a microRNA, *mir-786*, is responsible for restricting DMP pacemaker activity to the posterior-most intestinal cells by affecting lipid composition. In wild-type animals, the Ca^2+^ wave that initiates the DMP invariably begins in the posterior-most cells and spreads anteriorly [41]. In *mir-240*/*786* mutants, however, the wave frequently initiates in more anterior cells. In addition, the intensity and regularity of the wave is reduced in the mutants, and the period of the DMP increased. To explain these results, it has been proposed that *mir-786* acts in posterior cells to lower a threshold that they have for a factor that stimulates Ca^2+^ release [41]. This factor appears either to be a lipid itself or perhaps a lipid-modified protein. *mir-786*, which is strongly expressed in posterior intestinal cells, inhibits the translation or stability of mRNA from the *elo-2* gene, which encodes a fatty acid elongase that acts to lengthen the DMP cycle period [42]. Although *elo-2* is transcribed in all intestinal cells, its activity is downregulated in posterior cells by *mir-786*. ELO-2 catalyses the elongation of palmitate (C16:0) to stearate (C18:0) [42]. Thus, the increased threshold for initiation of the wave in posterior cells seen in *mir-240*/*786* mutants [41] could be the result of ELO-2-cataysed ectopic synthesis of stearate (or its derivatives) in posterior intestinal cells, reduced levels of ELO-2’s main substrate, palmitate, or a combination of these two effects. Notably, exogenously supplied palmitate rescues the increased cycle period seen in *mir-240*/*786* mutants [41] suggesting that palmitate reduces the threshold. Moreover, several mutants lacking the ability to make longer chain fatty acids have increased cycle periods rather than shorter [42]. Regardless of the exact mechanism, it is clear that lipids can affect the DMP. Our own observations imply that the DMP can, in turn, affect lipid uptake. From these findings, it would be tempting to speculate that dietary lipids might be important drivers for the DMP. It is known that nutrients can affect the DMP: the period is lengthened when worms are fed limiting amounts of food [43] or when feeding is disrupted by mutation [12]. Furthermore, worms that have left the bacterial lawn immediately cease to express the DMP [12]. However, although at first sight attractive, a model in which dietary nutrients alone drive the pacemaker is undoubtedly too simplistic: when worms leave the bacterial lawn on which they are feeding, the muscle contractions that are part of the DMP fail to occur but the period of the DMP is nevertheless often retained when they re-enter the lawn [43]. It is still possible, however, first, that dietary lipids might promote the proper expression of the DMP (*i*.*e*. the muscular contractions), or, second, that under some circumstances, they can influence the DMP cycle period. Consistent with this possibility, it has previously been shown that, although the muscular contractions are not required for the continued function of the pacemaker [12], both its period and consistency are reduced in constipated mutants [37, 43]. In particular, following an explosive defecation event in such mutants, the following cycle tends to be slightly longer than normal while subsequent cycles have progressively shorter periods becoming 30% shorter than wild type until the next explosive defecation. Our results suggest that it is possible that this shortening of the cycle period seen in certain DMP mutants might result, in part, from altered fat metabolism.

## Concluding Remarks

The DMP exists not just in *C*. *elegans* but in many other nematode species too, including some that are thought to have diverged from *C*. *elegans* well over a hundred million of years ago [44]. The DMP is thought, therefore, to have adaptive value. Since fatty acids are likely very important dietary nutrients for nematodes, a role for the DMP in promoting their uptake (or that of other nutrients) might help to explain why the program exists. Such a role also has important implications for studies of metabolism, growth control and nutrient-modulated behaviours in *C*. *elegans*. Worms are known to be able to adapt their feeding behaviour in response to altered internal metabolic state [35]. Our results suggest that worms might also be able to regulate internal nutrient levels by modulating the DMP.

## Supporting Information

S1 Fig *sv75* and *sv76* mutant animals do not have reduced numbers of lysosome-related organelles (LROs) in the intestine. Micrographs showing parts of the intestines of worms viewed with DIC (A-C) or fluorescence (D-F) optics. The animals in D-F were illuminated with blue light, which stimulates autofluorescence of LROs, one type of granule in the intestine. The mutants do not have reduced autofluorescence compared to wild type. G. Graph showing quantification of autofluorescence (in arbitrary units) from the intestines of adult hermaphrodites.
(TIF)

S2 Fig Digital images of Nile Red-stained worms analysed with Imaris software. The images on the left show a surface generated with the software corresponding to Nile Red-stained droplets in worms stained with the dye in the presence of isopropanol. Those on the right, show surfaces of the same worms generated with background fluorescence. The surfaces on the right allow the total volumes of the portions of the worms shown to be determined. Those on the left allow the total volume of the Nile Red-stained regions within these volumes to be determined. The surfaces together were used to determine the combined volumes of Nile Red-stained droplets per unit volume. To generate the graphs in Fig 1, for each genotype, two separate regions of six young adult worms lacking eggs were analysed in this way.
(TIF)

S3 Fig Pharyngeal pumping and function are not reduced in *aex-5* mutants; *aex-5* mutant dauer larvae are able to accumulate fat. A. Micrographs of dauer larval worms stained with Sudan Black viewed with DIC optics. *aex-5* mutant dauer larvae, like wild-type dauer larvae, accumulate fat. B. Graph showing the rate of pumping of the pharynx in wild-type animals and in and *aex-5* mutants. The rate of pharyngeal pumping was measured as previously described (Avery L (1993) The genetics of feeding in *Caenorhabditis elegans*. Genetics 133: 897–917). C. Fluorescence micrographs showing parts of the intestine of adult hermaphrodite worms fed with a mixture of bacteria and bacterium-sized fluorescent beads. The beads accumulate in the lumen of the intestine in both wild-type and mutant animals. The uptake of fluorescent beads was assayed as previously described (Kao G, et al. (2007) ASNA-1 positively regulates insulin secretion in *C*. *elegans* and mammalian cells. Cell 128: 577–587).
(TIF)

S4 Fig Other defecation mutants also have reduced steady state levels of fat in the intestine. A. Micrographs of 1-day old hermaphrodite worms stained with Oil Red O viewed with DIC optics. B. Micrographs of part of the intestines in 1-day old hermaphrodite worms stained with Sudan Black and viewed with DIC optics. In B, the outlines of the intestines are marked by dotted lines. Large white arrows indicate Sudan Black staining of the intestine. A subset of fertilized eggs stained with Sudan Black are indicated by small black arrows. Whereas in N2, staining is seen along the entire length of the intestine, in the *pbo*, *aex-4* and *egl-8* mutants, staining is restricted to the anterior part of the intestine. We have noticed that Sudan Black staining of the eggs is more intense in strains in which intestinal staining is reduced or lacking. C. Graph showing normalized ratios of total triglycerides to total phospholipids. Error bars denote 95% confidence intervals. * denotes a significant different in the means determined by one-way ANOVA and Fischer's test for least significant difference.
(TIF)

S5 Fig Defecation mutants display reduced staining with Nile Red. A. Fluorescence confocal micrographs of young adult hermaphrodite worms fixed with isopropanol and stained with Nile Red. B. Quantification of the Nile Red-stained regions with Imaris software. The regions were imaged and analysed by the methods described in the Materials and Methods section of the main text, and in the legend to S2 Fig. Error bars denote standard errors of the means. **** denotes significant difference in the means determined by one-way ANOVA and Dunnett's multiple comparisons test (σ = 0.05).
(TIF)
