# Supplementary figures and images for: Aberrant Fat Metabolism in Caenorhabditis elegans Mutants with Defects in the Defecation Motor Program
Source: PLoS One. 2015 Apr 7;10(4):e0124515. doi: 10.1371/journal.pone.0124515 (PMC4388766; doi:10.1371/journal.pone.0124515)

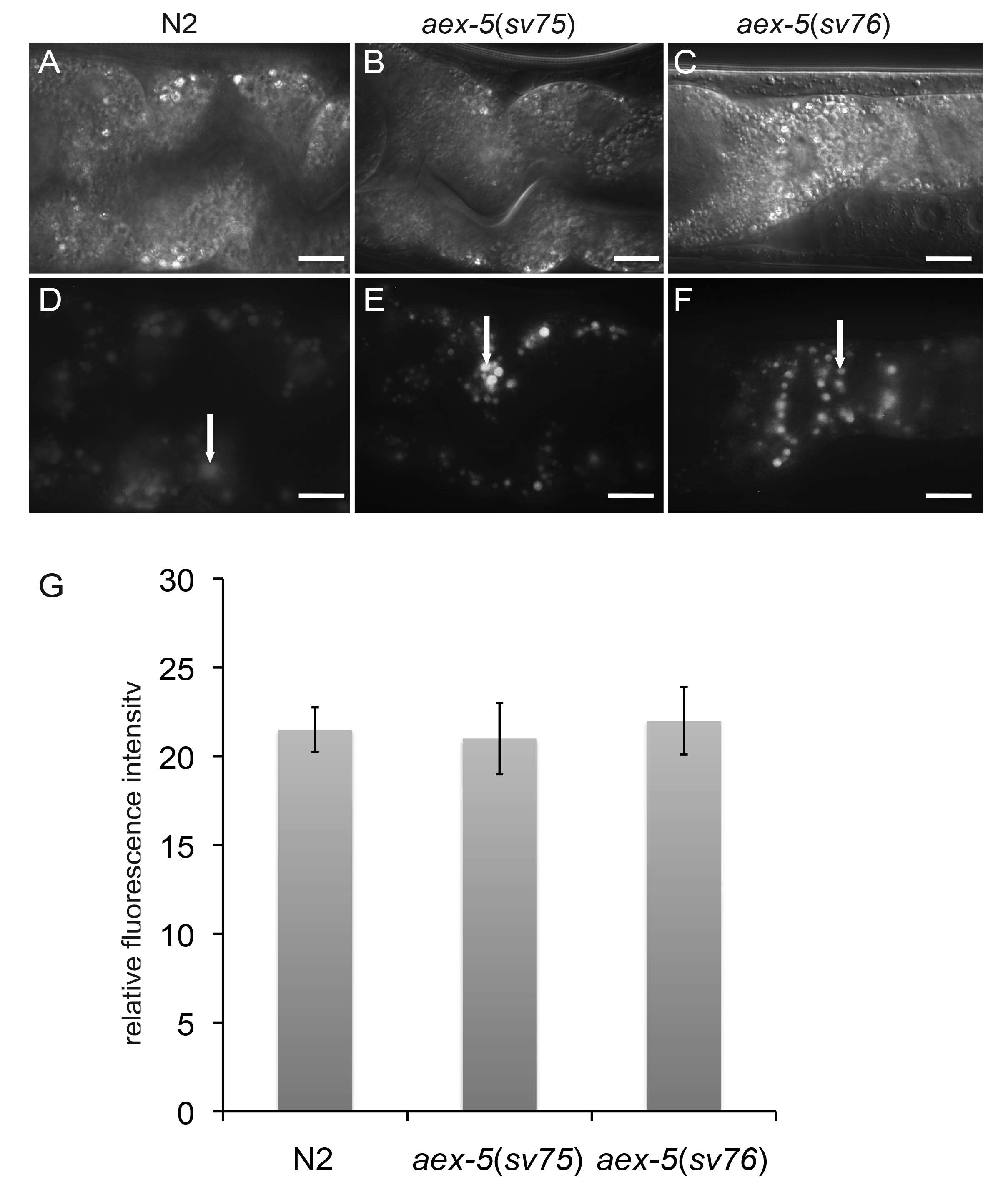

Supplement: S1 Fig — Micrographs showing parts of the intestines of worms viewed with DIC (A-C) or fluorescence (D-F) optics. The animals in D-F were illuminated with blue light, which stimulates autofluorescence of LROs, one type of granule in the intestine. The mutants do not have reduced autofluorescence compared to wild type. G. Graph showing quantification of autofluorescence (in arbitrary units) from the intestines of adult hermaphrodites. (TIF) [file pone.0124515.s001.tif]

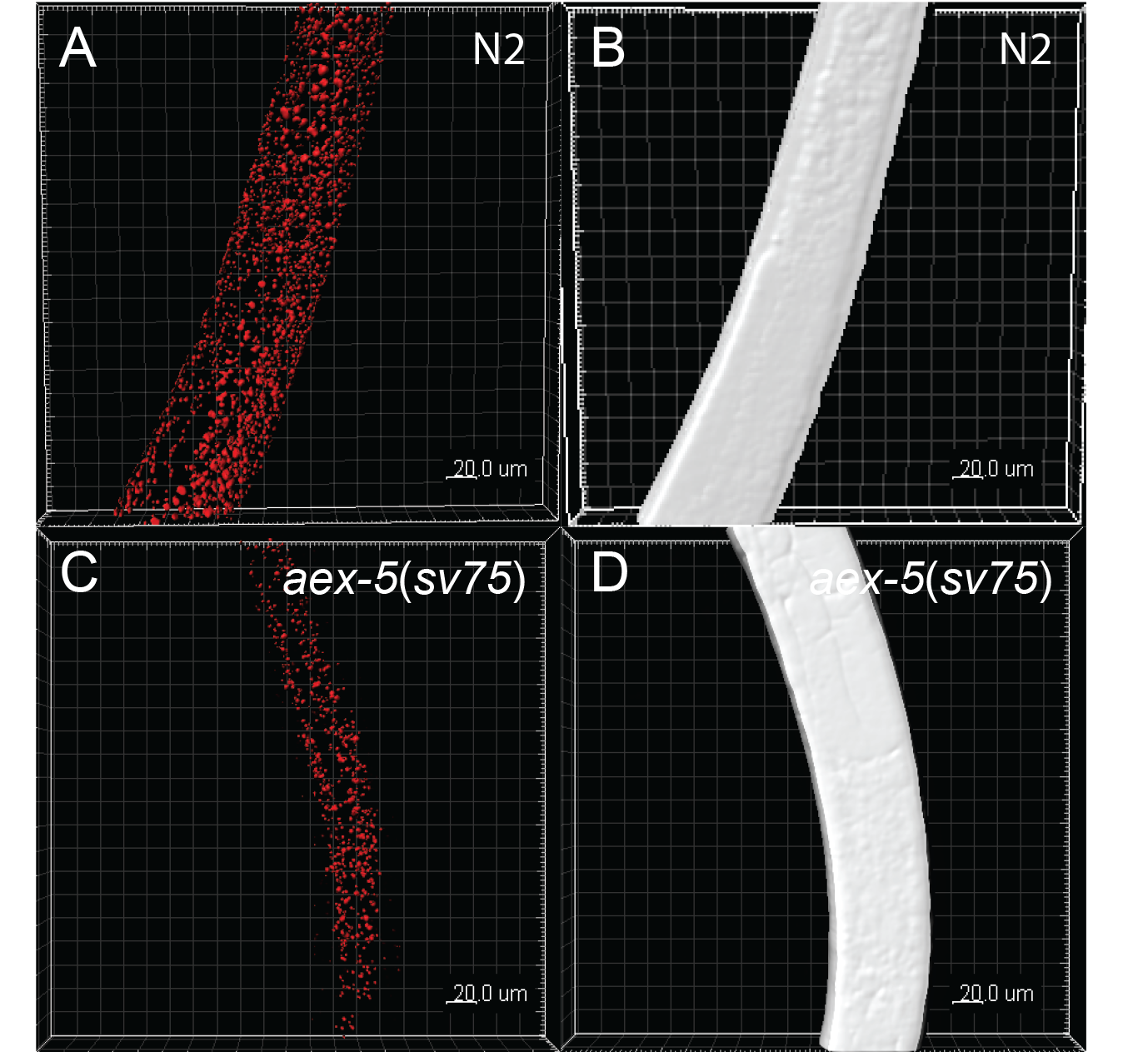

Supplement: S2 Fig — The images on the left show a surface generated with the software corresponding to Nile Red-stained droplets in worms stained with the dye in the presence of isopropanol. Those on the right, show surfaces of the same worms generated with background fluorescence. The surfaces on the right allow the total volumes of the portions of the worms shown to be determined. Those on the left allow the total volume of the Nile Red-stained regions within these volumes to be determined. The surfaces together were used to determine the combined volumes of Nile Red-stained droplets per unit volume. To generate the graphs in Fig 1, for each genotype, two separate regions of six young adult worms lacking eggs were analysed in this way. (TIF) [file pone.0124515.s002.tif]

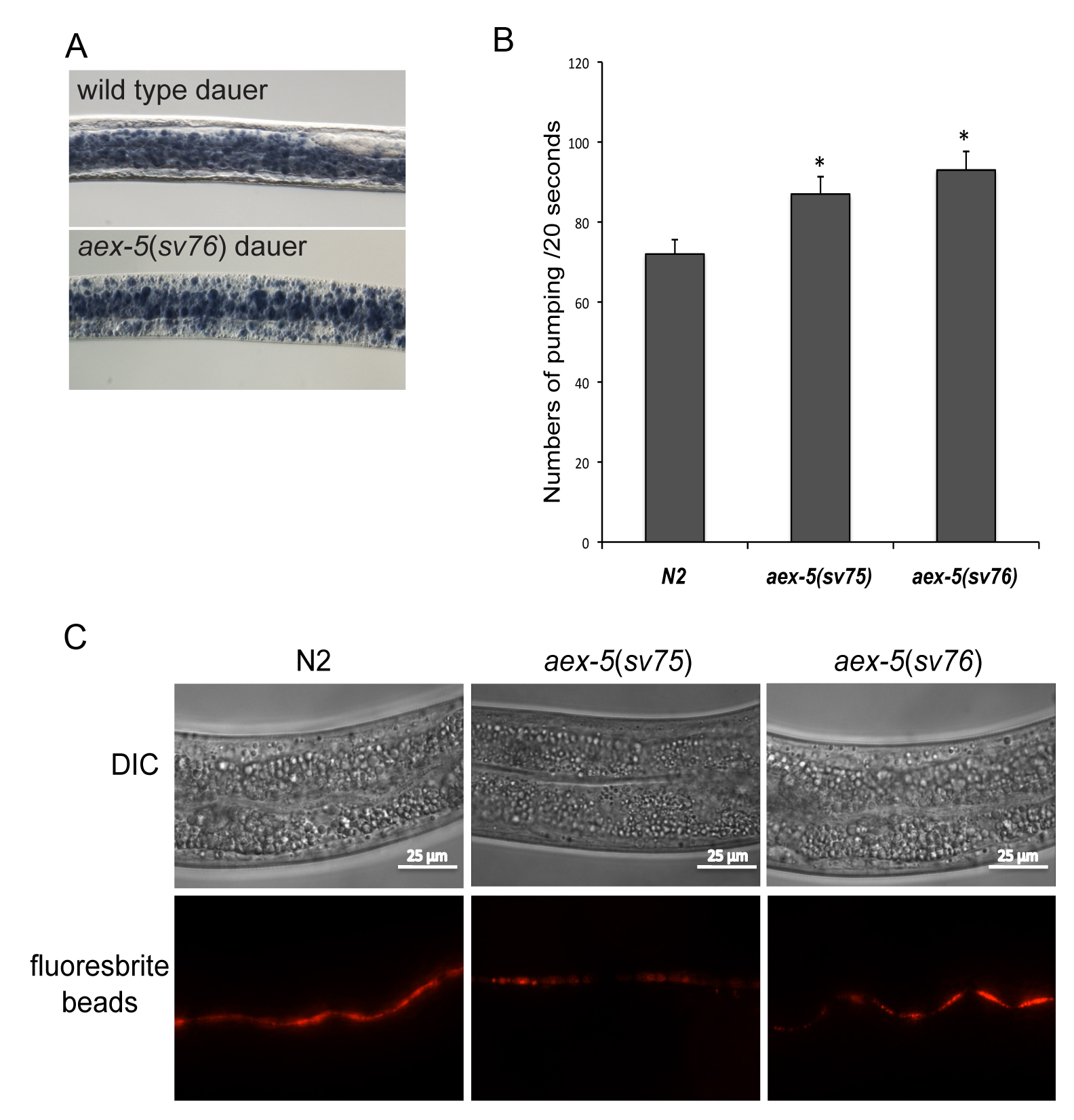

Supplement: S3 Fig — A. Micrographs of dauer larval worms stained with Sudan Black viewed with DIC optics. aex-5 mutant dauer larvae, like wild-type dauer larvae, accumulate fat. B. Graph showing the rate of pumping of the pharynx in wild-type animals and in and aex-5 mutants. The rate of pharyngeal pumping was measured as previously described (Avery L (1993) The genetics of feeding in Caenorhabditis elegans. Genetics 133: 897–917). C. Fluorescence micrographs showing parts of the intestine of adult hermaphrodite worms fed with a mixture of bacteria and bacterium-sized fluorescent beads. The beads accumulate in the lumen of the intestine in both wild-type and mutant animals. The uptake of fluorescent beads was assayed as previously described (Kao G, et al. (2007) ASNA-1 positively regulates insulin secretion in C. elegans and mammalian cells. Cell 128: 577–587). (TIF) [file pone.0124515.s003.tif]

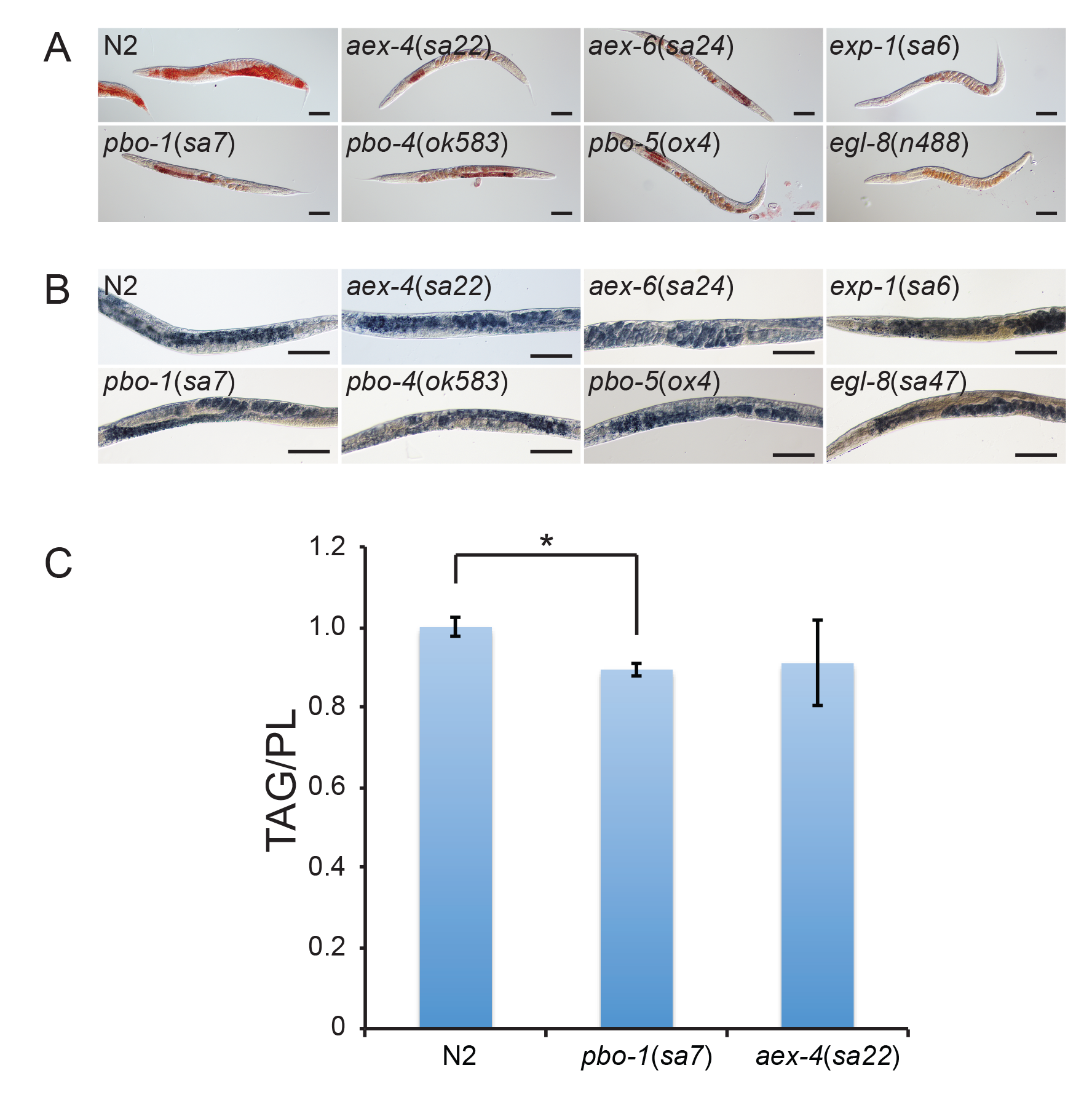

Supplement: S4 Fig — A. Micrographs of 1-day old hermaphrodite worms stained with Oil Red O viewed with DIC optics. B. Micrographs of part of the intestines in 1-day old hermaphrodite worms stained with Sudan Black and viewed with DIC optics. In B, the outlines of the intestines are marked by dotted lines. Large white arrows indicate Sudan Black staining of the intestine. A subset of fertilized eggs stained with Sudan Black are indicated by small black arrows. Whereas in N2, staining is seen along the entire length of the intestine, in the pbo, aex-4 and egl-8 mutants, staining is restricted to the anterior part of the intestine. We have noticed that Sudan Black staining of the eggs is more intense in strains in which intestinal staining is reduced or lacking. C. Graph showing normalized ratios of total triglycerides to total phospholipids. Error bars denote 95% confidence intervals. * denotes a significant different in the means determined by one-way ANOVA and Fischer's test for least significant difference. (TIF) [file pone.0124515.s004.tif]

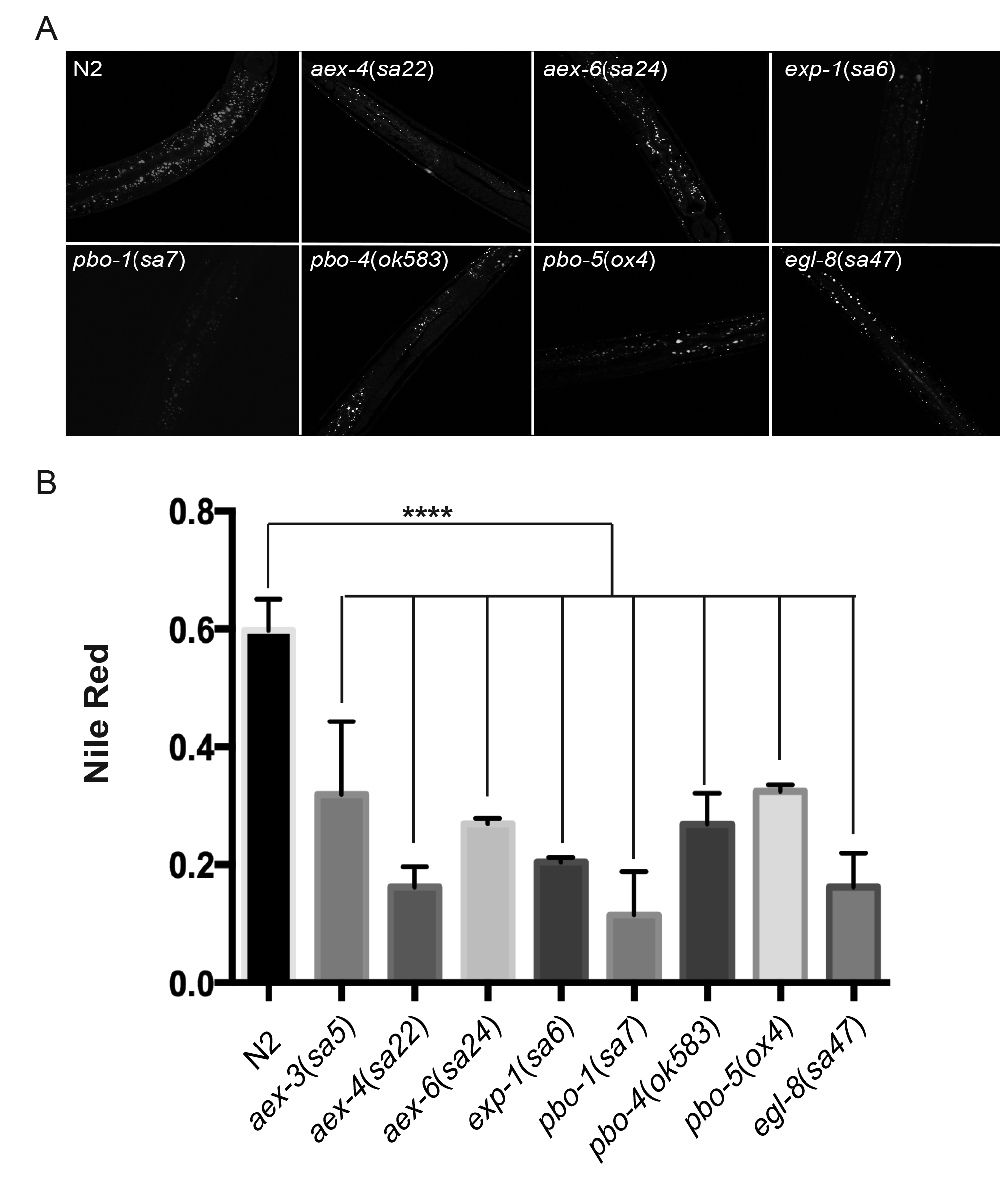

Supplement: S5 Fig — A. Fluorescence confocal micrographs of young adult hermaphrodite worms fixed with isopropanol and stained with Nile Red. B. Quantification of the Nile Red-stained regions with Imaris software. The regions were imaged and analysed by the methods described in the Materials and Methods section of the main text, and in the legend to S2 Fig. Error bars denote standard errors of the means. **** denotes significant difference in the means determined by one-way ANOVA and Dunnett's multiple comparisons test (σ = 0.05). (TIF) [file pone.0124515.s005.tif]
